# Supplementary material for: The Effectiveness of Planning Interventions for Improving Physical Activity in the General Population: A Systematic Review and Meta-Analysis of Randomized Controlled Trials
Source: Int J Environ Res Public Health. 2022 Jun 15;19(12):7337. doi: 10.3390/ijerph19127337 (PMC9223740; doi:10.3390/ijerph19127337)
Supplement: Supplementary file 1 [file ijerph-19-07337-s001.zip › Search strategy.pdf]

Pubmed strategy:

((("Exercise"[Mesh]) OR ((((((((((((((((((((((Physical Activity[Title/Abstract]) OR (Activities, Physical[Title/Abstract])) OR (Activity, Physical[Title/Abstract])) OR (Physical Activities[Title/Abstract])) OR (Exercise, Physical[Title/Abstract])) OR (Exercises, Physical[Title/Abstract])) OR (Physical Exercise[Title/Abstract])) OR (Physical Exercises[Title/Abstract])) OR (Acute Exercise[Title/Abstract])) OR (Acute Exercises[Title/Abstract])) OR (Exercise, Acute[Title/Abstract])) OR (Exercises, Acute[Title/Abstract])) OR (Exercise, Isometric[Title/Abstract])) OR (Exercises, Isometric[Title/Abstract])) OR (Isometric Exercises[Title/Abstract])) OR (Isometric Exercise[Title/Abstract])) OR (Exercise, Aerobic[Title/Abstract])) OR (Aerobic Exercise[Title/Abstract])) OR (Aerobic Exercises[Title/Abstract])) OR (Exercises, Aerobic[Title/Abstract])) OR (Exercise Training[Title/Abstract])) OR (Exercise Trainings[Title/Abstract])) OR (Training, Exercise[Title/Abstract])) OR (Trainings, Exercise[Title/Abstract]))) AND (("Planning Techniques"[Mesh]) OR ((((((((((((((((((((Planning Technique[Title/Abstract]) OR (Technique, Planning[Title/Abstract])) OR (Techniques, Planning[Title/Abstract])) OR (Planning Technic[Title/Abstract])) OR (Planning Technics[Title/Abstract])) OR (Technic, Planning[Title/Abstract])) OR (Technics, Planning[Title/Abstract])) OR (Methodology, Planning[Title/Abstract])) OR (Methodologies, Planning[Title/Abstract])) OR (Planning Methodologies[Title/Abstract])) OR (Planning Methodology[Title/Abstract])) OR (Planning Theories[Title/Abstract])) OR (Planning Theory[Title/Abstract])) OR (Theories, Planning[Title/Abstract])) OR (Theory, Planning[Title/Abstract])) OR (implementation intentions[Title/Abstract])) OR (intention of implementing[Title/Abstract])) OR (intention activation[Title/Abstract])) OR (planning techniques[Title/Abstract])) OR (implementation techniques[Title/Abstract])) OR (action planning[Title/Abstract])) OR (coping planning[Title/Abstract])))) AND (((randomized controlled trial[Publication Type]) OR (randomized[Title/Abstract])) OR (placebo[Title/Abstract]))

Embase strategy:

#1: 'physical activity'/exp

#2: 'exercise':ab,ti OR 'exercises':ab,ti OR 'physical activity':ab,ti OR 'activities, physical':ab,ti OR 'activity, physical':ab,ti OR 'physical activities':ab,ti OR 'exercise, physical':ab,ti OR 'exercises, physical':ab,ti OR 'physical exercise':ab,ti OR 'physical exercises':ab,ti OR 'acute exercise':ab,ti OR 'acute exercises':ab,ti OR 'exercise, acute':ab,ti OR 'exercises, acute':ab,ti OR 'exercise, isometric':ab,ti OR 'exercises, isometric':ab,ti OR 'isometric exercises':ab,ti OR 'isometric exercise':ab,ti OR 'exercise, aerobic':ab,ti OR 'aerobic exercise':ab,ti OR 'aerobic exercises':ab,ti OR 'exercises, aerobic':ab,ti OR 'exercise training':ab,ti OR 'exercise trainings':ab,ti OR 'training, exercise':ab,ti OR 'trainings, exercise':ab,ti

#3: #1 OR #2

#4: 'implementation intention'/exp

#5: 'planning technique':ab,ti OR 'technique, planning':ab,ti OR 'techniques, planning':ab,ti OR 'planning technic':ab,ti OR 'planning technics':ab,ti OR 'technic,

planning':ab,ti OR 'technics, planning':ab,ti OR 'methodology, planning':ab,ti OR 'methodologies, planning':ab,ti OR 'planning methodologies':ab,ti OR 'planning methodology':ab,ti OR 'planning theories':ab,ti OR 'planning theory':ab,ti OR 'theories, planning':ab,ti OR 'theory, planning':ab,ti OR 'implementation intentions':ab,ti OR 'intention of implementing':ab,ti OR 'intention activation':ab,ti OR 'planning techniques':ab,ti OR 'action planning':ab,ti OR 'coping planning':ab,ti
#6: #4 OR #5
#7: 'randomized controlled trial':ab,ti OR 'randomized':ab,ti OR 'placebo':ab,ti
#8: #3 AND #6 AND #7

Proquest strategy:
(mainsubject(physical activity) OR mainsubject(exercise))AND(ab((implementation intention) OR (action planning) OR (coping planning) OR (planning strategy) OR (planning intervention)))AND(ab((randomized controlled trial) or (randomized) or (placebo)))

Psycinfo strategy
(Abstract(physical activity) OR (exercise))AND(Abstract ((implementation intention) OR (action planning) OR (coping planning) OR (planning strategy) OR (planning intervention)))AND(Abstract ((randomized controlled trial) or (randomized) or (placebo)))

Web of Science strategy:
(TS=("Physical activity " OR Exercise OR Exercises OR "Physical Activity""Activities, Physical"OR"Activity, Physical"OR"Physical Activities"OR"Exercise, Physical"OR"Exercises, Physical"OR"Physical Exercise"OR"Physical Exercises"OR"Acute Exercise"OR"Acute Exercises"OR"Exercise, Acute"OR"Exercises, Acute"OR"Exercise, Isometric"OR"Exercises, Isometric"OR"Isometric Exercises"OR"Isometric Exercise"OR"Exercise, Aerobic"OR"Aerobic Exercise"OR"Aerobic Exercises"OR"Exercises, Aerobic"OR"Exercise Training"OR"Exercise Trainings"OR"Training, Exercise"OR"Trainings, Exercise"))AND(AB =("Planning techniques"OR"Planning Technique"OR"Technique, Planning"OR"Techniques, Planning"OR"Planning Technic"OR"Planning Technics"OR"Technic, Planning"OR"Technics,Planning"OR"Methodology, Planning"OR"Methodologies, Planning"OR"Planning Methodologies"OR"Planning Methodology"OR"Planning Theories"OR"Planning Theory"OR"Theories, Planning"OR"Theory, Planning"OR"implementation intentions"OR"intention of implementing"OR"intention activation" OR"planning techniques" OR"action planning" OR"coping planning"))AND(AB=("random* controlled trial" OR random* OR placebo))

Cochranelibrary strategy:
#1: MeSH descriptor: [Exercise] explode all trees

#2: (Exercise):ti,ab,kw OR (Exercises):ti,ab,kw OR (Physical Activity):ti,ab,kw OR (Activities, Physical):ti,ab,kw OR (Activity, Physical):ti,ab,kw OR (Physical Activities):ti,ab,kw OR (Exercise, Physical):ti,ab,kw OR (Exercises, Physical):ti,ab,kw OR (Physical Exercise):ti,ab,kw OR (Physical Exercises):ti,ab,kw OR (Acute Exercise):ti,ab,kw OR (Acute Exercises):ti,ab,kw OR (Exercise, Acute):ti,ab,kw OR (Exercises, Acute):ti,ab,kw OR (Exercise, Isometric):ti,ab,kw OR (Exercises, Isometric):ti,ab,kw OR (Isometric Exercises):ti,ab,kw OR (Isometric Exercise):ti,ab,kw OR (Exercise, Aerobic):ti,ab,kw OR (Aerobic Exercise):ti,ab,kw OR (Aerobic Exercises):ti,ab,kw OR (Exercises, Aerobic):ti,ab,kw OR (Exercise Training):ti,ab,kw OR (Exercise Trainings):ti,ab,kw OR (Training, Exercise):ti,ab,kw OR (Trainings, Exercise):ti,ab,kw
#3: #1OR #2
#4: (Planning Technique):ti,ab,kw OR (Technique, Planning):ti,ab,kw OR (Techniques, Planning):ti,ab,kw OR (Planning Technic):ti,ab,kw OR (Planning Technics):ti,ab,kw OR (Technic, Planning):ti,ab,kw OR (Technics, Planning):ti,ab,kw OR (Methodology, Planning):ti,ab,kw OR (Methodologies, Planning):ti,ab,kw OR (Planning Methodologies):ti,ab,kw OR (Planning Methodology):ti,ab,kw OR (Planning Theories):ti,ab,kw OR (Planning Theory):ti,ab,kw OR (Theories, Planning):ti,ab,kw OR (Theory, Planning):ti,ab,kw OR (implementation intentions):ti,ab,kw OR (intention of implementing):ti,ab,kw OR (intention activation):ti,ab,kw OR (planning techniques):ti,ab,kw OR (action planning):ti,ab,kw OR (coping planning):ti,ab,kw
#5: #3 AND #4

CNKI search strategy:
((((((主题%='身体活动' or 题名%='身体活动')OR(主题%='体力活动' or 题名%='体力活动'))OR(主题%='锻炼' or 题名%='锻炼'))OR(主题%='体育活动' or 题名%='体育活动'))AND(((((旧版主题='执行意向')OR(旧版主题='行动计划'))OR(旧版主题='应对计划'))OR(旧版主题='计划干预'))OR(旧版主题='计划策略')))AND(((((旧版主题='实验')OR(旧版主题='随机对照'))OR(旧版主题='随机分配'))OR(旧版主题='随机'))OR(旧版主题=xls('RCT'))));检索范围:期刊。

Wanfang data search strategy:
主题:(身体活动+体力活动+体育活动+锻炼) and 主题:(执行意向+行动计划+应对计划+计划干预+计划策略) and 主题:(随机+随机对照+随机分配+实验+RCT)
