# Supplementary figures and images for: The Effectiveness of Planning Interventions for Improving Physical Activity in the General Population: A Systematic Review and Meta-Analysis of Randomized Controlled Trials
Source: Int J Environ Res Public Health. 2022 Jun 15;19(12):7337. doi: 10.3390/ijerph19127337 (PMC9223740; doi:10.3390/ijerph19127337)

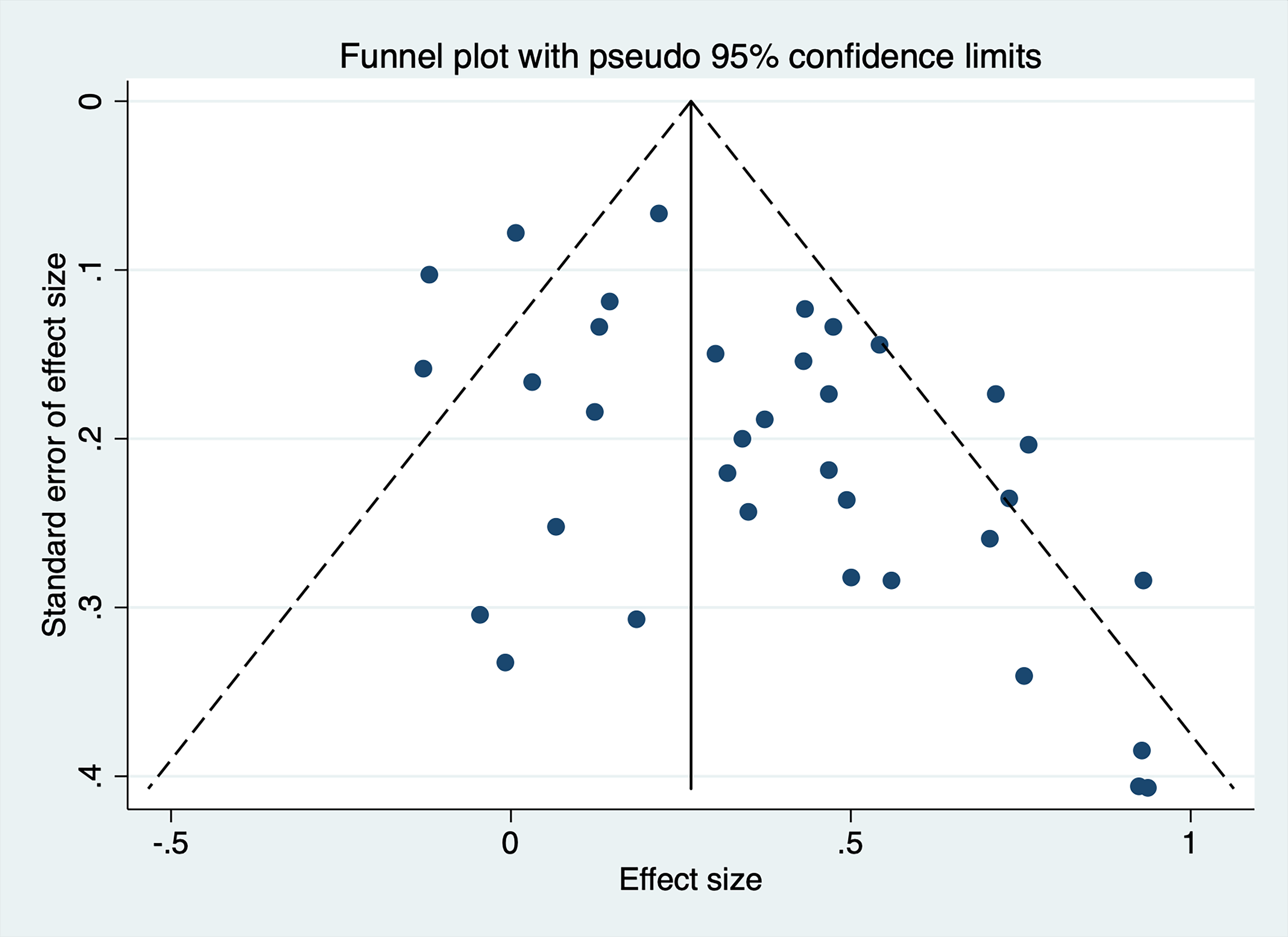

Supplement: Supplementary file 1 [file ijerph-19-07337-s001.zip › Figure S1 Funnel plot.tif]

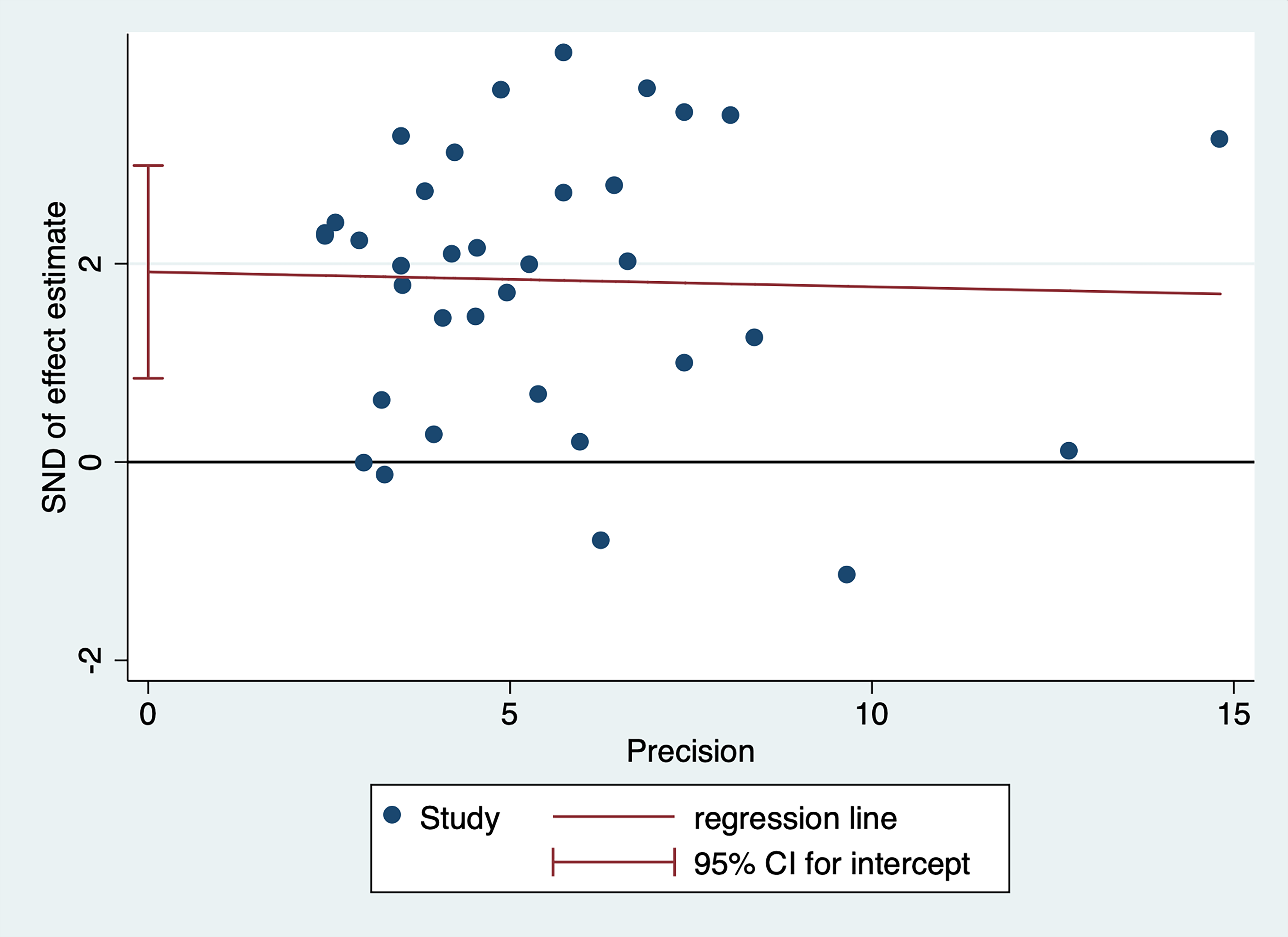

Supplement: Supplementary file 1 [file ijerph-19-07337-s001.zip › Figure S2 Egger plot.tif]
